# Confounding factors for the effect of misdiagnosis of site of infection on in-hospital mortality

**DOI:** 10.1186/s13054-019-2577-4

**Published:** 2019-09-03

**Authors:** Kei Suzuki, Shinichiro Ohshimo, Nobuaki Shime

**Affiliations:** Department of Emergency and Critical Care Medicine, Graduate School of Biomedical and Health Sciences, Hiroshima University, 1-2-3 Kasumi, Minami-ku, Hiroshima, 734-8551 Japan

To the Editor:

We would like to express our keen interest in the article published in a recent issue of *Critical Care* by Abe and colleagues [1], who investigated the effect of misdiagnosis or unidentified site of infection at initial examination on in-hospital mortality. The authors found that misdiagnosis of the initial site of infection was associated with a > 10% increase in in-hospital mortality among patients with infection. However, we believe that a number of factors potentially affecting the results of this study should be discussed.

Firstly, it is unclear whether the treatments for the initial diagnoses were appropriate. Although data regarding the site of infection and initial vital signs was provided, information about the duration before escalation and/or de-escalation to appropriate antibiotics according to the accurately diagnosed site of infection and pathogens was lacking. The information on appropriate fluid resuscitation and/or circulatory supports was also lacking. As delayed and inappropriate administration of antibiotics and insufficient fluid resuscitation have previously been associated with poor outcomes [2, 3], these factors may have affected the results of this study.

Secondly, it would be useful to have more information about the presence, if any, of antibiotic-resistant bacterial infections in this study. Infection with multidrug-resistant bacteria has been associated with poor outcomes [4], and the severity of drug resistance could vary between different tertiary hospitals. Therefore, this may confound the findings of this study.

Lastly, information on mixed etiology with multiple pathogens seems lacking. The authors defined patients according to the predominant pathogens, and complicated infections were not further defined. However, patients frequently suffer from multiple pathogens in the tertiary hospitals, which has previously been associated with poor outcomes [5]. Patients who were initially misdiagnosed in this study may have had a more complicated condition than the correctly diagnosed patients. Therefore, we speculate that mixed infection may be a confounding factor affecting the poor outcomes observed.

In conclusion, we believe that clarification of these issues by the authors would be helpful to better understand the effects of misdiagnosed site of infection at initial examinations on patient outcomes.

## Data Availability

Not applicable.
