# Supplementary material for: Confounding factors for the effect of misdiagnosis of site of infection on in-hospital mortality
Source: Crit Care. 2019 Sep 3;23:294. doi: 10.1186/s13054-019-2577-4 (PMC6720063; doi:10.1186/s13054-019-2577-4)
Supplement: Related texts

# Reply: Author’s response to letter “Confounding factors for the effect of misdiagnosis of site of infection on in-hospital mortality”

**Authors:** Toshikazu Abe

We thank Suzuki et al. for their interest in and comments regarding our study, which is a secondary analysis of an emergency room (ER) subset of the Sepsis Prognostication in Intensive Care Unit and ER (SPICE-ER) study from 2017 to 2018 [1]. As we mentioned, although the results of our study were limited, we obtained answers for some questions posed by Suzuki et al. using data from the Focused Outcomes Research in Emergency Care in Acute Respiratory Distress Syndrome, Sepsis, and Trauma (FORECAST) study conducted from 2016 to 2017 [6] because the SPICE-ER study included 35 emergency departments (EDs) in 59 FORECAST institutions, which had more data compared with the SPICE-ER study on their clinical practices of interest.

In the FORECAST study, carbapenem was most commonly used after initial diagnoses (55%) followed by tazobactam/piperacillin (21%) and vancomycin (18%) [6]. Most patients (84%) received broad-spectrum antibiotics within 3 h of sepsis recognition. However, we found no information on the escalation or de-escalation of antibiotics. Few studies have focused on antibiotic stewardship in patients with sepsis. In the FORECAST study, approximately 90% of patients achieved sepsis care bundle 2012 [7] about supportive therapy with fluid resuscitation and vasopressors for sepsis. Our microbiologic blood culture results did not include data on antibiotic-resistant pathogens. Despite this, few patients were probably infected by antibiotic-resistant pathogens because we exclusively chose patients from EDs for the SPICE study [1]. Even if antibiotic-resistant pathogens were present, the most important pathogens would have been covered by the chosen antibiotics. Complicated patients may have had multiple infection sites and mixed pathogens. However, empirical treatments consider possible entry sites, infected sites, and predominant pathogens even in the absence of confirmed infections such as primary bacteremia. Therefore, the influence of confounding factors which Suzuki et al. indicated may have been small.

Diagnosing the infection site is crucial for reasons other than ensuring the right antibiotic choice [1, 6], and further studies need to focus on this issue. Our results suggest that time-sensitive treatments only with broad-spectrum antibiotics may warrant reconsideration. Moreover, renewing focus on ensuring precise diagnosis for choosing more appropriate antibiotics and improving subsequent treatments including resuscitation, adjunctive therapies, and definitive therapies is essential.
